# Supplementary material for: Children’s Representations of Possibility: Uncertainty About “What” Is Easier Than Uncertainty About “Where”
Source: Open Mind (Camb). 2026 Jul 15;10:951–62. doi: 10.1162/OPMI.a.364 (PMC13421556; doi:10.1162/OPMI.a.364)
Supplement: Supplementary file 1 [file opmi-10-951-s001.pdf]

Children's representations of possibility: Uncertainty about "what" is easier than uncertainty about "where"

Esra Nur Turan-Küçük and Melissa M. Kibbe

Supplement

Participants Supplement

Of the n = 2 children who declined to complete study procedures in the Uncertain Locations condition, 1 child stopped after test trial 2, 1 child stopped after test trial 3.

Of the n = 5 children who declined to complete study procedures in the Uncertain Identities condition, 2 children declined to complete the first familiarization trial, 2 children stopped after familiarization trial 1, and 1 child stopped after test trial 1.

Table S1. Comparisons of children's mean proportion correct across trials to guessing (.333) for each age year.

	mean	*t*	*p*	*Cohen's d*
Uncertain Locations				
2-year-olds	.57	3.73	.002	.88
3-year-olds	.64	6.36	<.001	1.29
4-year-olds	.77	7.29	<.001	1.49
Uncertain Identities				
2-year-olds	.67	9.32	<.001	1.90
3-year-olds	.85	13.17	<.001	2.69
4-year-olds	.85	12.31	<.001	2.51

<u>Additional main model details</u>

Although Trial Number was not retained in the best-fitting model, we tested whether including Trial Number altered the significance of our factors of interest. We fit a generalized linear mixed-effects model (GLMM) with a binomial link function, including Age, Condition, and Trial Number, with random intercepts for Subject.

Including Trial Number did not change the results. Age remained a significant positive predictor of performance ($\beta = 0.038$, $SE = 0.015$, $z = 2.50$, $p = .013$), and children continued to perform

significantly better in the Uncertain Identities condition than in the Uncertain Locations condition ($\beta = 0.77$, $SE = 0.23$, $z = 3.26$, $p = .001$). Trial Number itself was not a significant predictor ($p = .18$), and including it did not improve model fit ($\chi^2(1) = 1.76$, $p = .18$).

We also tested the Condition × Age interaction by comparing the base model to a model additionally including the interaction term. The Condition × Age interaction was not significant ($\beta = 0.022$, $SE = 0.023$, $z = 0.95$, $p = .343$), and model comparison confirmed that adding the interaction did not improve model fit ($\chi^2(1) = 0.89$, $p = .34$).

<u>*Are children learning across test trials?*</u>

To examine whether children's performance changed systematically across the four Test trials (i.e., whether there was evidence of learning or practice effects), we fit a trial-level logistic mixed-effects model predicting accuracy from Trial Number. Trial Number was coded 1–4 and mean-centered. The model included random intercepts for Subject (Response ~ Trial_c + (1 | Subject). Trial Number was not a significant predictor of accuracy ($\beta = -0.12$, $SE = 0.09$, $z = -1.32$, $p = .19$).

<u>*Order effects*</u>

We conducted a *post hoc* exploratory analysis to examine whether children's responses varied depending on whether the target cup (i.e., the cup adjacent to the revealed cup) was in the set of cups that was occluded first or last. To do so, we divided the data based on whether the revealed cup (and therefore the correct response) was in the first-hidden set or the last-hidden set, and ran a mixed effects logistic regression with Condition (Identity vs. Location), Hidden Order (First Hidden vs. Last Hidden), Age (Continuous, Centered), all two-way interactions between Condition, Hidden Order, and Age, and the three-way interaction (Condition × Hidden Order × Age) as fixed effects, and participant as a random intercept. We found significant main effect of Condition ($\beta = -1.16$, $SE = 0.32$, $z = -3.67$, $p < .001$), a significant main effect of Age ($\beta = 0.066$, $SE = 0.024$, $z = 2.72$, $p = .006$), no significant main effect of Hidden Order ($\beta = -0.23$, $SE = 0.32$, $z = -0.72$, $p = .473$). The Condition × Hidden Order interaction did not reach significance ($\beta = 0.80$, $SE = 0.43$, $z = 1.87$, $p = .061$); inspection of the data suggested that children in the Uncertain Location condition did slightly better when the revealed cup was in the last-hidden set compared to the first-hidden set, while children in the Uncertain Identities condition did similarly well across the board. Neither the Condition × Age interaction ($\beta = -0.003$, $SE = 0.031$, $z = -0.11$, $p = .916$) nor the Hidden Order × Age interaction ($\beta = -0.014$, $SE = 0.032$, $z = -0.45$, $p = .655$) reached significance, indicating that the effect of condition on performance did not vary with age, and the three-way Condition × Hidden Order × Age interaction was also non-significant ($\beta = -0.029$, $SE = 0.042$, $z = -0.69$, $p = .489$). While the condition x hidden order interaction did not reach significance, the trend in the data suggest that future work is needed to examine whether maintaining representations of uncertain locations may be more demanding than maintaining representations of uncertain identities.

Could condition differences be driven by children using different lower-level strategies across conditions?

We argue that the condition differences we observed in our study are explained by the representational range of children's object indexing architecture. Here we considered a potential alternative explanation for our results: if children were using a strategy of avoiding the "shown empty" side in the Uncertain Locations condition, and selecting the adjacent cup in the "shown non-target" side in the Uncertain Identities condition, this would result in a pattern of greater success in the Uncertain Identities condition and worse performance in the Uncertain Locations condition[1].

To examine this possibility, we considered a few possible ways in which such a lower-level strategy would play out across age, and whether these scenarios fit the pattern we observed in our data:

We first considered a possible scenario in which children's lower-level strategy use increases with age. In our data, children's success at the task in *both* conditions increased with age. That is, we observed a main effect of condition, and a main effect of age, with no age x condition interaction. Children's increasing success in the Uncertain Identities condition with age could potentially be due to increasing use of the lower-level strategy to pick an adjacent cup, since such a strategy would result in more correct responses. But if that is the case, we should also expect that children's use of this strategy would increase in the Uncertain Locations condition. This would mean that children should show *decreasing* success with age, as the strategy of avoiding the "shown empty" side would lead to an incorrect response. Instead, we observe increasing success in both conditions across age.

We then considered a possible scenario in which a consistent subset of children exhibit the lower-level strategies across age. If a consistent proportion of children across our age range are using these lower-level strategies, while the remainder of the children either succeed or fail without using the strategy (depending on age), this subset of children could be driving the main effect of condition. If this is the case, we should observe similar proportions of *all correct* and *almost all correct* responses in the Uncertain Identities condition, for which the bias strategy always yields the correct answer, and *all incorrect* or *almost all incorrect* responses in the Uncertain Locations condition, for which the bias strategy always yields the incorrect answer. However, children rarely are all incorrect or mostly incorrect in the Uncertain Locations condition (see Table S2), suggesting there is not a proportion of children consistently deploying the "avoid empty side" strategy in that condition.

[1] We thank an anonymous reviewer for raising this possibility.

Table S2. Distribution of mean proportion correct scores in the Uncertain Locations and Uncertain Identities conditions.

Proportion correct	Uncertain Locations	Uncertain Identities
**0**	2	0
**.25**	6	1
**.5**	24	17
**.75**	14	23
**1**	20	31

We then considered a third possible scenario: children may exhibit asymmetrical strategy use. Perhaps children only use a lower-level strategy in the Uncertain Identities condition - that is, maybe children use a “pick adjacent” strategy in the Uncertain Identities condition, and there is no corresponding “avoid empty” strategy in the Uncertain Locations condition. If this is the case, and some proportion of children are consistently using such a strategy across our age range, this might yield higher performance in the Uncertain Identities condition across age, without a corresponding decrease in performance in the Uncertain Locations condition.

To examine this possibility (and as a further test of whether children are deploying any of these strategies in either condition), we utilized the data from our third familiarization trial, in which children were tasked with finding a target in one of three cups. Specifically, in our three-cup familiarization trial, children were shown two sets of cups, a singleton set and a doubleton set. In the Uncertain Locations condition, a sticker was hidden inside the singleton set and another sticker was hidden inside one of the cups in the doubleton set (with location uncertain). In the Uncertain Identities condition, a smiley face eraser was hidden inside the singleton set, and a smiley face eraser and a block hidden inside the cups in the doubleton set (with identity uncertain). Children then were asked to find a target object. The correct response is to choose the singleton cup in both conditions, since it surely contains the target. Since we did not reveal the contents of any of the cups during this trial, a strategy of “avoid empty side” or “select adjacent cup”, respectively, would not be possible.

With the necessary caveats that we did not design our experiment *a priori* to adjudicate lower-level strategy use, and we did not plan this analysis in advance so it should be considered exploratory, we examined whether performance on the 4-cup test trials systematically differed from the 3-cup familiarization trial in a way that would be predicted by the lower-level strategy explanation (i.e., in the Uncertain Identity condition, children should do better in the 4-cup trials than the 3-cup trial, and/or in the Uncertain Locations condition, children should do worse in 4-cup trials than the 3-cup trial). To do so, we included children’s responses on the 3-cup familiarization trial as the first “test” trial in the sequence (i.e., the reference level), followed by test trials 1–4. We fit a generalized linear mixed-effects model predicting accuracy (logit scale) from

centered age (in months), condition (uncertain locations vs. uncertain identities), their interaction, and trial number (modeled as a factor), and a condition x trial number interaction, with random intercepts for subjects. Consistent with our planned analyses reported in the main paper, there was a significant main effect of age ($\beta = 0.039$, SE = 0.012, $z = 3.14$, $p = .002$). A likelihood ratio test confirmed a significant main effect of condition; children performed better in the Uncertain Identities condition than in the Uncertain Locations condition ($\chi^2(1) = 11.13$, $p < .001$). The age x condition interaction was not significant ($\beta = 0.003$, SE = 0.019, $z = 0.17$, $p = .87$). Critically, none of the condition X trial interaction terms were significant (all ps > .21), and model comparison confirmed that adding the condition x trial interaction did not improve the model fit ($\chi^2(4) = 2.17$, $p = .705$). This suggests that children's responses in the 3-cup familiarization trial did not differ systematically from their responses in subsequent test trials as a function of condition.

Finally, we note that the pattern of results that we observed in our study is consistent with patterns observed in previous studies for which children the use of these specific lower-level strategies does not apply. Three previous papers with children showed that 3-year-olds succeed at possibility reasoning tasks that involve mutually-exclusive possible identities (Turan-Küçük & Kibbe, 2025a, 2025b, see also Alderete & Xu, 2023) earlier than similar tasks that involve mutually-exclusive possible locations, and even infants show evidence of representing mutually-exclusive possible identities for a partially-hidden object whose identity is ambiguous (Cesana-Arlotti et al. 2012, 2018, 2022). Brody et al. (2024) found that adults, when tasked with tracking objects into occlusion, had more difficulty when there was uncertainty about the objects' locations compared to when there was uncertainty about the objects' identities. We suggest that our results, interpreted in this larger context, reveal real differences in the way children are representing the objects in these tasks.
